# Differences in chronic spontaneous urticaria between Europe and Central/South America: results of the multi-center real world AWARE study

**DOI:** 10.1186/s40413-018-0216-1

**Published:** 2018-11-16

**Authors:** M. Maurer, K. Houghton, C. Costa, F. Dabove, L. F. Ensina, A. Giménez-Arnau, G. Guillet, G. N. Konstantinou, M. Labrador-Horrillo, H. Lapeere, R. Meshkova, E. A. Pastorello, M. Velásquez-Lopera, L. M. Tamayo Quijano, C. Vestergaard, N. Chapman-Rothe

**Affiliations:** 1 Department of Dermatology and Allergy, Charité - Universitätsmedizin Berlin, Charitéplatz 1, 10117 Berlin, Germany; 2 RTI Health Solutions, Carolina, North, USA; 3 Serviço de Imunoalergologia, Hospital de Santa Maria, Lisbon, Portugal; 4 Center of Medical Specialties Lobos, Buenos Aires, Argentina; 5 CPAlpha Clinical Research Center, São Paulo, Brazil; 6 Hospital Sírio-Libanês, São Paulo, Brazil; 7 Hospital del Mar, Parc de Salut Mar, IMIM, Universitat Autònoma de Barcelona, Barcelona, Spain; 8 Service de Dermato-allergologie, CHU Poitiers, Poitiers, France; 9 Department of Allergy and Clinical Immunology, 424 General Military Training Hospital, Thessaloniki, Greece; 10 Hospital Vall d’Hebron, VHIR, Universitat Autònoma de Barcelona, Barcelona, Spain; 11 Department of Dermatology, Ghent University Hospital, Ghent, Belgium; 12 Allergy and Clinical Immunology, Smolensk State Medical University, Smolensk, Russia; 13 S.C. di Allergologia e Immunologia ASST Grande Ospedale Metropolitano Niguarda Milan, Milan, Italy; 14 Centro de Investigaciones Dermatológicas, CIDERM, University of Antioquia, Medellín, Colombia; 15 Pontificia Bolivariana University, Medellín, Colombia; 16 Department of Dermatology and Venereology, Aarhus University Hospital, Aarhus, Denmark; 17 Novartis Pharma AG, Basel, Switzerland

**Keywords:** Angioedema, Chronic spontaneous urticaria, Quality of life, Urticaria

## Abstract

**Background:**

Global chronic urticaria (CU) disease experience and management is not well documented. This study descriptively compares these aspects among CU patients residing in Europe (EU) and Central and South America (C/SA).

**Methods:**

AWARE (A World-wide Antihistamine-Refractory chronic urticaria patient Evaluation) is a global prospective, non-interventional study of CU in the real-world setting. Patients were ≥ 18 years with a diagnosis of H1-antihistamine-refractory CU for > 2 months. Differences between the EU and C/SA regions in demographic and clinical characteristics, quality of life (QoL), work and activity impairment, pharmacological treatment, and healthcare resource use were examined.

**Results:**

In total, 4224 patients were included in the analysis (C/SA 492; EU 3732). Rates of untreated patients were greater in the C/SA region (45.1% vs. 31.9%; *P* < 0.005) and escalation to third-line therapy was rare in both regions. Differences in disease experience emerged, with C/SA patients more commonly experiencing angioedema (C/SA 50.8% vs. EU 46.1%; *P* = 0.03) or comorbid chronic inducible urticaria (C/SA 30% vs. EU 22%; *P* < 0.001). Correspondingly, rates of uncontrolled urticaria were higher among C/SA patients (82.8% vs. 77.5%; *P* = 0.017) and patients in the C/SA region showed significantly greater work and activity impairment (absenteeism: 10.4 ± 19.7 vs. 6.7 ± 19.0, *P* = 0.004; presenteeism: 30.3 ± 31.9 vs. 24.4 ± 25.8, *P* = 0.001; work productivity loss: 33.9 ± 33.9 vs. 26.5 ± 27.5, *P* < 0.001; activity impairment: 37.7 ± 34.7 vs. 32.7 ± 30.1, *P* = 0.001). However, QoL impairment was greater in the EU region (Dermatology Life Quality Index: C/SA 6.5 ± 5.9 vs. EU 8.3 ± 7.0; *P* < 0.001). There was a significant difference in use of healthcare resources, including emergency services (39.6% vs. 29.3%; *P* < 0.001), hospitalization (7.7% vs 21.9%; *P* < 0.001) general practitioners (31.7% vs 57.3%; *P* < 0.001), and additional allergists or dermatologists (50.6% vs. 47.3%, *P* < 0.001), among patients in the C/SA and EU region, respectively. In both regions, patients with a primary diagnosis of CU with angioedema had significantly greater impairment in work and non-work activities and healthcare resource utilization compared to those without angioedema.

**Conclusions:**

This study revealed that CU is a heterogeneous condition with differences in healthcare utilization and outcomes between EU and C/SA. However, overall there is a high unmet need of H1-antihistamine-refractory CU patients, which is associated with high use of healthcare resources, and has a large negative effect on QoL and work productivity.

## Background

Chronic urticaria (CU) is a skin disorder in which red, swollen, itchy, and sometimes painful hives (wheals), angioedema, or both, repeatedly occur for more than 6 weeks [1]. Hives may occur with or without angioedema (swelling in the deep layers of the skin that causes itch, pain or burning), particularly around the eyes, lips, cheeks, hands, feet, and genitals [2]. Likewise, recurrent angioedema may occur with or without wheals. The estimated prevalence of CU is up to 1% in the general population [3], with those aged between 30 and 50 years most commonly affected, and females affected approximately twice as often as males [4–7]. A further distinction is made between urticarias with spontaneously occurring signs and symptoms – chronic spontaneous urticaria (CSU) – and those where wheals and angioedema are induced by specific stimuli (including cold, heat, solar, delayed pressure, vibratory, dermographic, aquagenic, cholinergic, contact, and exercise – chronic inducible urticaria (CIndU) [8–11]. Patients may concurrently experience CSU and CIndU in approximately 20% of cases [5].

While the condition is not life threatening, the impact of the disorder on patient quality of life, work productivity, and activities of daily living have been well documented [12–14]. To improve symptom control and minimize disease burden among CU patients, international guidelines have been developed, which recommend a stepwise treatment approach [1]. Initial treatment should consist of labelled doses of second-generation non-sedating H1-antihistamines (sgH1-AH). Where an inadequate response is observed after 2–4 weeks or earlier if symptoms are intolerable, up-titration (up to four times) of the H1-AH dose is recommended. If inadequate symptom relief persists after 4 weeks of the higher dose H1-AH, the previous version of the EAACI/GA^2^LEN/EDF/WAO guidelines (relevant at the time of the AWARE study) recommended add-on treatment with montelukast, omalizumab, or ciclosporin [15, 16]. The 2017 version of the guideline recommends omalizumab as third line treatment and ciclosporin A as fourth line treatment [1], although no recommendations are made regarding the length of time that such treatment should be administered. Available literature suggests that the guideline recommendations have not been consistently followed, leading to an unmet need within the CU population [12, 17–21].

The majority of published data on H1-refractory CU is limited to patient populations derived from specialized urticaria centers and thus is not representative of the general population of patients with CU. To collect data from a representative sample of CU patients across the world, the AWARE (a World-wide Antihistamine-Refractory chronic urticaria patient Evaluation) non-interventional, prospective observational study was designed. The AWARE study assesses therapy regimens, burden of disease, and healthcare resource utilization among H1-antihistamine refractory adult CU patients treated by either office-based dermatologists and allergists or specialized urticaria centres. Baseline results of the AWARE study have been reported for Germany [13] and Scandinavia [22]. The prevalence and demographic composition is thought to be similar across the world, but the differences and similarities in disease experience and the effects of the disease are not well documented. Here, we use the baseline results of the AWARE study to assess the differences and similarities in the experience of urticaria by comparing two patient populations that differ culturally, specifically in terms of health care systems. Thus, we compare data among the included countries across the European Union (EU) region and those across the Central and South America (C/SA) region. Reported data include population characteristics, disease activity, disease subtype, angioedema rates, pharmacological treatment, disease-specific quality of life (QoL), work productivity and activity impairment, and rates of healthcare resource utilization.

## Methods

### Patients and study design

AWARE was a prospective observational non-interventional study that followed CU patients for 2 years, refractory to the approved dose of at least one H1-antihistamine. The present study reports on patients who were enrolled in urticaria centres and office-based dermatological and allergological practices between March 2014 and April 2016. Clinics that indicated interest in participating were visited prior to study commencement.

To be included within the study, patients had to meet the following criteria: a medically confirmed diagnosis of CU that had been present for at least 2 months; refractory to treatment with H1-antihistamine; at least 18 years of age; able and willing to provide informed consent. Patients were excluded from the study if they were participating in a clinical trial, or if difficulties were anticipated with patient follow-up.

A total of 4232 H1-antihistamine refractory CSU patients were enrolled from 458 sites worldwide, including Belgium, Germany, Denmark, Spain, France, the United Kingdom, Greece, Italy, Norway, Portugal, Russia, Sweden, Argentina, Brazil, Colombia, Costa Rica, the Dominican Republic, Guatemala, Honduras, Panamá, and Perú. A total of 4224 patients met the inclusion criteria (*n* = 8 were excluded: *n* = 4 patients were missing data on age and n = 4 were aged < 18 years and thus did not meet inclusion criteria), comprised of 3732 patients from the EU and 492 patients from the C/SA region. Table 1 shows the number of eligible patients recruited within each country.

**Table 1:** Patient distribution within each country

		Number of recruited, eligible patients
Region EU	Belgium	80
	Germany	2254
	Denmark	82
	Spain	277
	France	92
	United Kingdom	264
	Greece	145
	Italy	249
	Norway	50
	Portugal	76
	Russia	135
	Sweden	28
Region C/SA	Argentina	96
	Brazil	100
	Colombia	77
	Costa Rica	24
	Dominican Republic	54
	Guatemala	30
	Honduras	51
	Panama	25
	Peru	35

### Outcome measures

During the first visit, the following information was collected and analysed: demographics (age, gender, height, and weight); clinician-reported diagnosis (CSU and CIndU, with or without angioedema); disease control, measured by the Urticaria Control Test (UCT [23]); patient-reported quality of life, measured by the Dermatology Life Quality Index (DLQI [24]); patient-reported work and activity impairment, measured by the Work, Productivity, and Activity Impairment questionnaire (WPAI [25]); clinician-reported current and prior (within the past 12 months, but not currently received) pharmacological treatment for CU; patient-reported satisfaction with treatment, measured on a Visual Analogue Scale (VAS) ranging from 0 (totally unsatisfied) to 10 (completely satisfied); and, clinician-reported frequency of healthcare resource use due to urticaria (from the time of first report of CU symptoms).

The UCT is a disease-specific measure consisting of four questions that retrospectively assesses patients’ burden of disease over the previous 4 weeks. Concepts covered include disease activity, QoL, disease control, and therapy. A total score from 0 to 16 points is derived, with a score of ≥12 indicating disease control [23, 26].

The DLQI is a dermatologic-specific QoL instrument consisting of 10 items covering six domains: symptoms and feelings, daily activities, leisure, work and school, personal relationships, and treatment [27]. A total score from 0 to 30 points is derived, and can be banded in the following ways: 0–1 no effect of CU on QoL; 2– moderate effect of CU on QoL; 6–10 large effect of CU on QoL; 11–20 very large effect of CU on QoL; and, 21–30 extremely large effect of CU on QoL [28].

The WPAI was used to evaluate work productivity and activity impairment due to chronic urticaria with the aid of six short questions relating to absenteeism (the percentage of work time missed due to urticaria), presenteeism (the percentage of impairment experienced while at work), overall work productivity (an overall work impairment estimate based on absenteeism and presenteeism) and overall activity impairment (the percentage of impairment in daily activities) over the previous 7 days. Only employed patients were asked to respond to the work-related questions, whereas all patients respond to the activity-related questions.

### Statistical analysis

Data derived from countries within the EU region were pooled and compared against pooled data from countries within the C/SA region. Means, medians, standard deviation (SD), minimum and maximum are stated for quantitative measurements and absolute and relative frequencies for categorical measurements. Tests for statistically significant differences at the 5% level (i.e., *P* < 0.05) were applied, with one-way analysis of variance (ANOVA) used to compare differences in means, and chi^2^ tests of distribution used to compare differences in sample distribution for categorical variables. The Bonferroni correction was applied to all conducted ANOVA tests, to reduce the chances of type I error which may arise when multiple pair-wise tests are performed.

Due to the observational nature of the study, incomplete data was present for some variables. No statistical missing data management strategies (e.g. imputation) were applied. Where missing data were present for categorical response variables the proportion of patients with missing data was included, unless otherwise noted. All analyses were conducted using the statistical software packages Stata (version 14) or SAS (version 9.2).

## Results

### CSU patients in central and South America are more likely to be young and female than CSU patients in Europe

Patients in the C/SA region were significantly younger (42.0 ± 13.8 vs. 46.0 ± 15.5; *P* < 0.001) and more likely to be female (79.3% vs. 70.8%; *P* < 0.001) compared with patients in the EU region (Table 2).

**Table 2:** Baseline demographic and clinical characteristics of H1- antihistamine-refractory CU patients

	Region EU	Region C/SA	Significance Test Statistic
Age (years)	46.0 ± 15.5	42.0 ± 13.8	*F* = 29.57; *P* < 0.001
Female: n (%)	2642 (70.8)	390 (72.3)	Chi^2^ = 15.350; *P* < 0.001
BMI (kg/m^2^)^a^	26.8 ± 5.4	26.3 ± 4.2	*F* = 1.58; *P* = 0.209
Underweight: n (%)	43 (1.3)	4 (1.7)	Chi^2^ = 5.436; *P* = 0.143
Healthy weight: n (%)	1361 (40.9)	99 (40.7)	
Overweight: n (%)	1157 (34.8)	98 (40.3)	
Obese: n (%)	764 (23.0)	42 (17.3)	
Diagnosis: n (%)			
CSU only	2725 (73.1)	303 (65.3)	Chi^2^ = 17.285; *P* = 0.001
CIndU only	215 (5.8)	31 (6.7)	
Both CSU and CIndU	756 (20.3)	129 (27.8)	
CSU with angioedema	1721 (46.1)	250 (50.8)	Chi^2^ = 6.839; *P* = 0.03
Time since diagnosis (years)	4.8 ± 7.2	3.0 ± 4.9	*F* = 23.33; *P* < 0.001
UCT score^b^	7.8 ± 4.3	7.2 (4.1)	*F* = 5.86; *P* = 0.016
UCT scores < 12: n (%)^b^	2277 (77.5)	391 (82.8)	Chi^2^ = 6.867; *P* = 0.009

*CIndU* chronic inducible urticaria; *CSU* chronic spontaneous urticaria; *UCT* urticaria control test

^a^BMI data was available for 3325 patients in the EU region and 243 patients in the C/SA region: the presented proportions are based on these denominators

^b^UCT data was available at visit 1 for 2939 patients in the EU region and 472 patients in the C/SA region: the presented proportions are based on these denominators

### CSU patients in central and South America are more likely to also have CIndU

**Fig. 1:**
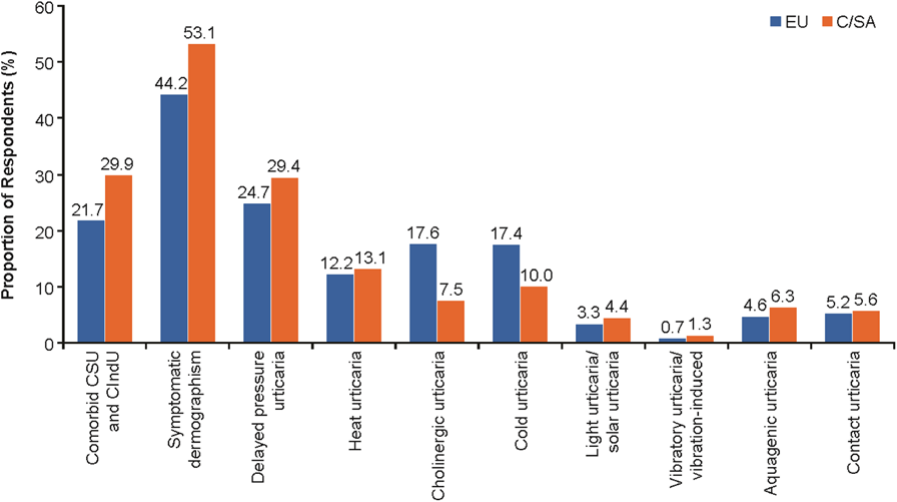
Frequency of CIndU experience

Among patients with CSU, CIndU was a comorbid disease in 30% of C/SA patients but only 22% of EU patients (*P* < 0.001). CSU patients in the C/SA region were significantly more likely to have comorbid symptomatic dermographism than those in the EU region (*P* = 0.04, Fig. 1). CSU patients in the EU region were significantly more likely to have cholinergic (*P* = 0.001) or cold urticaria (*P* = 0.02).

### CSU patients in central and South America have shorter time since diagnosis than patients in Europe

Within the C/SA region, the mean time since diagnosis in patients with CSU-only and patients with comorbid CSU and CIndU was 2.8 ± 4.8 and 3.7 ± 5.0 years, respectively, which was significantly shorter than in the EU region (4.6 ± 7.1 and 5.4 ± 7.0 years, *P* = 0.02 and 0.03, respectively).

### Angioedema occurs more often in CSU patients in central and South America

A significantly greater proportion of C/SA vs. EU patients had angioedema (50.8% vs. 46.1%; *P* = 0.03). Angioedema had occurred during the previous 6 months in 47.4% of C/SA patients vs. 42.6% of EU patients (*P* = 0.03). However, angioedema was rated more severely by patients in the EU: severe, moderate, mild, and negligible angioedema, respectively, were reported by 22.9%, 43.1%, 30.1%, and 2.7%, of EU patients vs. 12.9%, 45.9%, 40.8%, and 0% of patients in the C/SA region (*P* < 0.001).

### CSU patients in central and South America show lower rates of controlled disease than patients in Europe

Patients in the C/SA region had significantly lower UCT scores than those in Europe (7.2 ± 4.1 vs. 7.7 ± 4.3; *P* = 0.02), with a significantly greater proportion of patients in the C/SA region experiencing uncontrolled urticaria (UCT score < 12) compared to EU patients (82.8% vs. 77.5%; *P* = 0.01).

### CU patients in central and South America show higher rates of no treatment than patients in Europe

Rates of untreated patients (i.e., patients refractory to the approved dose of at least one H1-antihistamine, who were not currently receiving treatment) were high in both regions, but C/SA patients were significantly more likely to go untreated (45.1% vs. 31.9%; *P* < 0.005). Patients in the C/SA region were also significantly less likely to have been prescribed a therapy for CU within the previous 12 months (which was discontinued prior to the baseline visit) than patients in the EU region (31.1% vs. 42.3%; *P* < 0.001).

### CU patients in central and South America are less likely to receive omalizumab treatment than patients in Europe

Among those receiving treatment, the recommended first-line therapy for CU (i.e., sgH1-AH) were most commonly prescribed (77.4% EU vs. 86.1% C/SA; *P* = 0.001). Escalation to third-line therapy with omalizumab had occurred in significantly more EU patients than C/SA patients (21.0% vs. 11.4%; *P* < 0.001), while escalation to montelukast had occurred in significantly more C/SA patients than EU patients (11.8% vs. 6.1%; *P* < 0.001). Ciclosporin prescription was rare and comparable in both populations, with current receipt by 1.2% of patients in the EU region and 1.4% of patients in the C/SA region (*P* = 0.983). Current corticosteroid use was documented in 12.7% of patients receiving therapy in the EU region vs. 8.6% in the C/SA region (*P* = 0.05). The use of first generation H1-antihistamines was more prevalent in the C/SA region (21.1% vs. 13.8%; *P* = 0.07). Among those receiving a prior therapy, first generation H1-antihistamines had been received by 21.2% of EU patients vs. 34.6% of C/SA patients (*P* < 0.001).

### CU patients in central and South America show lower quality of life impairment than patients in Europe

Patients in the EU region had significantly higher DLQI scores than those in the C/SA region, indicating a greater effect of CU on QoL (8.3 ± 7.0 vs. 6.5 ± 5.9; *P* < 0.001); 32.2% of patients in the EU region reported a very large or extremely large effect of CU on QoL vs. 15.3% of patients in the C/SA region; 22.2% vs. 16.3%, respectively, reported a moderate impact (*P* < 0.001).

### CU-driven work impairment is greater in patients in central and South America

Among patients who were currently employed (57.5% in the EU region and 56.1% in the C/SA region), the mean (SD) estimated proportion of work time missed (i.e. absenteeism), percentage impairment while working (i.e. presenteeism) and the percentage overall work impairment (i.e. work productivity loss) was significantly greater for patients in the C/SA region compared to those in the EU region (10.4 ± 19.7 vs. 6.7 ± 19.0, *P* = 0.004; 30.3 ± 31.9 vs. 24.4 ± 25.8, *P* = 0.001; and, 33.9 ± 33.9 vs. 26.5 ± 27.5, *P* < 0.001, respectively). The estimated percentage of activity impairment among H1-antihistamine-refractory CU patients was also significantly greater for C/SA patients compared to EU patients (37.7 ± 34.7 vs. 32.7 ± 30.1, *P* = 0.001).

In both regions, patients with a primary diagnosis of CU with angioedema had significantly greater impairment in work and non-work activities compared to those without angioedema (Table 3).

**Table 3:** DLQI and WPAI scores among patients with a primary diagnosis of CSU with or without angioedema

Region EU				Region C/SA		
PRO	CSU with angioedema (*n* = 1721)	CSU without angioedema (*n* = 1760)	Significance Test Statistic	CSU with angioedema (*n* = 250)	CSU without angioedema (*n* = 182)	Significance Test Statistic
DLQI: mean (SD)	9.1 (7.5)	7.6 (6.3)	*F* = 37.28; *Ρ* < 0.001	7.0 (6.4)	5.9 (5.5)	*F* = 2.23; *P* = 0.137
No effect	17.8%	16.3%	Chi^2^ = 65.413; *Ρ* < 0.001	24.7%	24.3%	Chi^2^ = 4.4596; *P* = 0.347
Small effect	22.5%	29.5%		27.3%	33.1%	
Moderate effect	20.7%	23.7%		21.3%	25.0%	
Very large effect	28.0%	23.2%		22.0%	15.5%	
Extremely large effect	9.5%	4.5%		4.7%	2.0%	
WPAI Absenteeism: mean (SD)	9.4 (22.6)	4.4 (14.8)	*F* = 30.72; *Ρ* < 0.001	14.2 (23.6)	5.0 (12.7)	*F* = 11.67; *P* = 0.001
WPAI Presenteeism: mean (SD)	27.1 (27.5)	22.4 (24.2)	*F* = 14.5; *Ρ* < 0.001	35.3 (33.2)	22.3 (28.9)	*F* = 9.56; *P* = 0.002
WPAI Work Productivity Loss: mean (SD)	29.6 (29.4)	24.2 (25.8)	*F* = 16.82; *Ρ* < 0.001	39.9 (24.9)	24.3 (30.7)	*F* = 11.63; *P* = 0.001
WPAI Activity Impairment: mean (SD)	36.6 (31.9)	29.2 (28.1)	*F* = 47.14; *Ρ* < 0.001	41.1 (36.2)	31.3 (31.3)	*F* = 7.99; *P* = 0.005

### CU patients in central and South America show different healthcare resource utilization than patients in Europe

Since CU diagnosis, a significantly greater proportion of patients in the C/SA region had visited an emergency room department due to urticaria than those in the EU region (39.6% vs. 29.3%; *P* < 0.001). Additionally, patients in the C/SA region had a higher frequency of visits to emergency departments, 5.1 (15.1) vs. 3.5 (7.0), respectively; (*P* = 0.005). However, hospitalizations due to urticaria were significantly more likely to occur among patients from the EU region versus those from the C/SA region (21.9% vs. 7.7%; *P* < 0.001) and patients in the EU region were more frequent in the number of hospitalizations (2.0 [3.1] versus 0.3 [1.7], respectively; *P* < 0.001).

Similarly, visits to a general practitioner due to urticaria since diagnosis were significantly more common among patients from the EU region versus those from the C/SA region (57.3% vs. 31.7%; *P* < 0.001), with an average of 7.8 (14.9) versus 1.7 (4.0) visits (*P* < 0.001), respectively. In contrast, patients in the C/SA region were significantly more likely to visit a dermatologist or allergist versus those in the EU region (50.6% vs. 47.3%, *P* < 0.001), but the average number of visits was non-significantly greater in the EU region (7.5 [24.1] vs. 5.0 [14.9], *P* = 0.05). Other healthcare resources were used due to urticaria since diagnosis by a significantly greater proportion of patients in the EU region versus those in the C/SA region (38.6% vs. 19.7%, *P* < 0.001).

In both the C/SA and EU regions, patients with a primary diagnosis of CSU with angioedema had a significantly higher rate of healthcare resource utilization compared to those without angioedema (Table 4). This difference was particularly evident in terms of visits to an emergency room and hospitalizations.

**Table 4:** Healthcare resource use since diagnosis among patients with a primary diagnosis of CSU with or without angioedema

EU Region				C/SA Region		
Healthcare Resource	CSU with angioedema (*n* = 1721)	CSU without angioedema (*n* = 1760)	Significance Test Statistic	CSU with angioedema (*n* = 250)	CSU without angioedema (*n* = 182)	Significance Test Statistic
Emergency room						
Proportion of patients	40.4%	20.0%	Chi^2^ = 175.474; *Ρ* < 0.001	54.0%	25.8%	Chi^2^ = 38.379; *P* < 0.001
Frequency of visits	4.0 (8.1)	2.5 (4.1)	*F* = 9.70; *Ρ* = 0.002	8.0 (19.0)	2.1 (8.9)	*F* = 13.42; *P* < 0.001
Hospitalizations						
Proportion of patients	29.1%	15.7%	Chi^2^ = 90.023; *Ρ* < 0.001	11.4%	5.2%	Chi^2^ = 4.913; *P* = 0.027
Frequency of visits	2.3 (3.7)	1.5 (1.2)	*F* = 9.69; *Ρ* = 0.002	0.4 (2.1)	0.2 (1.3)	*F* = 1.86; *P* = 0.173
General practitioner						
Proportion of patients	61.9%	53.5%	Chi^2^ = 24.977; *Ρ* < 0.001	38.1%	32.2%	Chi^2^ = 1.547; *P* = 0.214
Frequency of visits	9.5 (18.4)	5.8 (9.0)	*F* = 27.72; *Ρ* < 0.001	2.3 (5.0)	1.1 (2.5)	*F* = 8.34; *P* = 0.004
Dermatologist or allergist						
Proportion of patients	53.2%	41.2%	Chi^2^ = 50.552; *Ρ* < 0.001	54.2%	54.0%	Chi^2^ = 0.002;*P* = 0.966
Frequency of visits	7.9 (26.6)	7.0 (22.3)	*F* = 0.41; *P* = 0.523	5.3 (11.3)	5.9 (20.6)	*F* = 0.13; *P* = 0.715

## Discussion

This analysis of baseline data collected as part of the AWARE study reveals that the clinical presentation of H1-antihistamine refractory CU and its impact on QoL and healthcare resource utilization is heterogeneous. Patients residing in the EU region compared with those in the C/SA region are less likely to experience angioedema and comorbid CSU and CIndU, have higher rates of controlled disease and treatment (including higher rates of escalation to omalizumab) and have lower rates of work impairment. However, EU patients report a greater effect of their condition on QoL than C/SA patients. Heterogeneity in healthcare resource use was also evidenced, with EU patients more likely to visit a general practitioner and be hospitalized, and less likely to visit dermatologist or allergists, or emergency departments.

A portion of the heterogeneity in disease severity may be explained by the observed differences in both demographic characteristics and treatment. Patients in the C/SA region were younger females who had been diagnosed for a shorter period of time. The observed higher rates of uncontrolled disease and impact of disease upon activity and work impairment may be linked to the lower rates of treatment, particularly escalation to omalizumab. Indeed, discrepancies in awareness of the current treatment guidelines have been recently evidenced, with physicians in Ecuador (18%) exhibiting lower awareness compared to physicians in Germany (33%) [17]. The higher levels of QoL reported in the C/SA region are not a new phenomenon: prior research has shown this region to have inexplicably high QoL evaluations [29].

Within the present observational study conducted across the EU and C/SA regions, the demographic characteristics are comparable to those previously reported within discrete countries: patients were predominantly female, overweight, with a mean age of 42 years in the EU region and 46 years in the C/SA region, and a preponderance of patients within the 18–40 age bracket [30–32]. Interestingly, the present study found that patients in the C/SA region were more likely to be older females than those in the EU region. The frequency of CSU and CIndU comorbidity has not yet been well documented; this study indicates that the comorbidity rate in CSU patients who are refractory to H1-antihistamines is approximately 20% among patients within the EU region and 28% around patients in the C/SA region. It is critical in clinical practice to recognize the possibility of such a co-occurrence, to ensure that patients are adequately tested for potential urticaria triggers and allow an individualized, personalized treatment approach. Although there is no distinct treatment algorithm specific to CIndU (that is, the guidelines recommend the same treatment approach as that for CSU), it is known that sgH1-AH do not reduce symptoms in all cases, and while up-titrating the dose leads to further improvement within these patients, it does not always lead to complete symptom relief [33–35]. Studies and case reports suggest that improvements similar to those observed with CSU can be achieved with omalizumab [10, 11, 36, 37].

Previous studies have shown angioedema rates among patients with CSU anywhere in the range from 33 to 85% [38–40]: in this multi-location study spanning Europe, Central America, and South America we identified that 46% of EU region patients and 51% of C/SA region patients experienced angioedema, with intensity ratings that were predominantly of moderate or severe intensity. It has been speculated that the varying rate of angioedema diagnosis among CU patients may be due to variability in physician diagnoses and the lack of consistently applied criteria [41]. Impairment in QoL, work, and non-work activities were greater for patients with angioedema compared to those without. This suggests that it is critical to manage the angioedema associated with CU, to reduce the patient burden. Additionally, rates of healthcare resource utilization were found to be much greater for patients with angioedema, particularly in terms of emergency room visits and hospitalizations. This supports the claim made by Staubach et al. [35] that it is important to educate patients that angioedema in CSU patients is not life-threatening and can be managed by dermatologists, allergists, or primary care physicians.

The current urticaria guidelines recommend that complete symptom control should be the aim of CU treatment. Findings from this observational study suggest that this goal is currently far from being achieved: UCT scores evidenced that more than three quarters of the recruited sample had uncontrolled urticaria despite the average presence of symptoms for 5 years or more. This issue was found to be significantly more prominent in the C/SA region. According to the 2013 EAACI/GA^2^LEN/EDF/WAO guidelines (relevant at the time of the AWARE study), first-line treatment with sgH1-AH is recommended, followed by an increase in dose (up to 4-fold) as second-line treatment and finally the addition of omalizumab, ciclosporin, or montelukast to the sgH1-AH as third-line treatment. [16] At the time of baseline documentation, 31.9% of patients in the EU region and 43.1% of patients in the C/SA region were currently not receiving pharmacological treatment (ranging from March 2014 for the earliest recruited patient to April 2016 for the last recruited patient), despite being diagnosed for a number of years, lack of urticaria control, a large effect of the condition of QoL, low treatment satisfaction, and high healthcare resource utilization. Moreover, the prescription of the recommended third-line treatment to achieve complete symptom control was present in less than 21% of the EU population and less than 12% of the C/SA population, and yet 76% and 83% of the populations, respectively, reported uncontrolled urticaria. No data was collected on the attribution for lack of treatment, because it was unanticipated during study design that treatment rates would be so low. However, recent research suggests that clinicians are not always following urticaria treatment guidelines [17, 20, 42] and patients may have essentially ‘given up’ in favor of self-treatment or simply living with the condition [41]. Another possible reason for non-medication or non-escalation to third-line treatment could be the differences in coverage and payment for healthcare among EU and C/SA region. For example, omalizumab is unavailable in some countries or its cost is high and not covered by health insurance programs (for example, in South America) [43]. In Ecuador, systemic steroids and first generation antihistamines are cheaper than sgAHs [17]. Economic considerations are an important and decisive factor for the choice of treatment.

Perhaps unsurprisingly, the rate of emergency healthcare resource use was high. However, it ought to be recognized that the AWARE study does not allow for an assessment of current healthcare resource use in general. This is particularly pertinent considering the findings from the cross-sectional web-based survey of patients diagnosed with CU in Germany, which revealed that only 40% of symptomatic patients were under physician care: the majority had stopped consulting a physician [41]. Similar results have been reported in Italy [42]. Thus, the burden of CU may be greater than currently estimated, if a substantial proportion of patients have stopped consulting healthcare professionals despite continuation of symptoms. The low rates of treatment satisfaction and high rates of uncontrolled urticaria found within the present study may be associated with this issue and further investigation is warranted.

Interestingly, within the EU region patients were consulting primary care physicians for their urticaria at a higher rate than that for dermatologists and allergists. This finding is novel: previous studies have reported that patients primarily consult with dermatologists or allergists about their condition [30, 42]. Thus, it is important to recognize that primary care physicians are a crucial part of the treatment team within the EU region and ensuring physicians are educated in line with the guideline recommendations is necessary.

Overall, across both the EU and C/SA regions, chronic urticaria is largely uncontrolled, under treated, is associated with a high healthcare resource use burden, and has a large effect on quality of life, work, and activity. Among patients who are treated, escalation to third-line therapies as recommended within the treatment guidelines is rare, despite the lack of disease control, low rates of patient satisfaction with treatment, and frequency of healthcare resource use. Further, this study identified that patients residing within the C/SA region versus the EU region have a greater uncontrolled symptom burden, a greater effect of CU on work, productivity, and activity, and a lower overall treatment rate. Additionally, patients within the C/SA region have a greater incidence of urticaria induced in response to a specific stimuli than patients in the EU region, with the latter more likely to experience spontaneous urticaria. This study also revealed that CU within the C/SA region is associated with a significantly greater incidence of emergency room visits and a significantly lower incidence of general practitioner visits. This suggests a potential benefit from greater education efforts at both the physician and patient level within the C/SA region. The differences identified between EU and C/SA region although statistically significant and relevant on a population level, do not prove that these regional differences are clinically significant for patients as individuals. An investigation of the individual clinical significance of the differences identified in this study requires longitudinal data, which should be the focus of further investigations. Potential reasons for the differences between regions include differences in how physicians approach CSU, e.g. differences in awareness of the need for symptomatic treatment that results in control of CSU and the absence of signs and symptoms, differences in the availability of treatment options, as well as differences in economic resources and funding for these options. It is likely that, in addition, differences in how patients approach CSU are related to the differences identified, e.g. differences in how satisfied patients are with partial response, differences in the availability of treatment and whether patients can afford to pay for it, as well as cultural differences in the perception of the burden of disease. Our results should encourage physicians, independent of the regions they work in, to aim for a reduction of non-treatment and under-treatment in their patient populations. The objectives of management, together with the means available to achieve these objectives, should be clearly discussed with patients, i.e., controlled disease and absence of signs and symptoms.

Strengths of the present study include the inclusion of patients across Europe, South America, and Central America, which allows for a multi-national understanding of the current urticaria situation. New insight is provided in to the disease burden at both the personal and societal level and current adherence to treatment guidelines. The study is limited due to the dependence on physicians and patients for data completeness and quality as well as the physicians’ free choice to enrol patients with CU refractory to regular doses of H1-antihistamines, independently of disease severity or medication needed to control symptoms and signs. Efforts were made at each site to encourage physicians to ensure that data records were filled in completely and accurately.

## Conclusions

This initial baseline analysis of the AWARE study reveals a high unmet need of H1-antihistamine refractory CU patients. Critically, this study revealed that CU is a heterogeneous condition with differences in healthcare utilization and outcomes between Europe and Central and South America. Further research is warranted to investigate the condition globally and identify region-specific features that may be used to inform public health strategies. There is a need for improved patient care, physician education, and adherence to current treatment guidelines, alongside harmonization of the management of CU globally, such that patients are receiving the highest standard care available based on the most current knowledge. The development of a world-wide network of urticarial centers of reference and excellence, alongside a global urticarial registry, may help to achieve this.

## Abbreviations

ANOVA: One-way analysis of variance
C/SA: Central and South America region
CIndU: Chronic inducible urticaria
CSU: Chronic spontaneous urticaria
CU: Chronic urticaria
DLQI: Dermatology Life Quality Index
EU: European Union
H1-AH: H1-antihistamines
QoL: Quality of life
SD: Standard deviation
UCT: Urticaria Control Test
VAS: Visual Analogue Scale
WPAI: Work, Productivity, and Activity Impairment
